# Copper in colorectal cancer: From copper‐related mechanisms to clinical cancer therapies

**DOI:** 10.1002/ctm2.1724

**Published:** 2024-05-28

**Authors:** Yuhong Wang, Pei Pei, Kai Yang, Lingchuan Guo, Yuan Li

**Affiliations:** ^1^ Department of Pathology Fudan University Shanghai Cancer Center Shanghai China; ^2^ Department of Oncology Shanghai Medical College Fudan University Shanghai China; ^3^ Department of Pathology The First Affiliated Hospital of Soochow University Suzhou China; ^4^ State Key Laboratory of Radiation Medicine and Protection School of Radiation Medicine and Protection & School for Radiological and Interdisciplinary Sciences (RAD‐X) Collaborative Innovation Center of Radiation Medicine of Jiangsu Higher Education Institutions Soochow University Suzhou Jiangsu China

**Keywords:** cancer therapy, colorectal cancer, copper, cuproptosis

## Abstract

Copper, a trace element and vital cofactor, plays a crucial role in the maintenance of biological functions. Recent evidence has established significant correlations between copper levels, cancer development and metastasis. The strong redox‐active properties of copper offer both benefits and disadvantages to cancer cells. The intestinal tract, which is primarily responsible for copper uptake and regulation, may suffer from an imbalance in copper homeostasis. Colorectal cancer (CRC) is the most prevalent primary cancer of the intestinal tract and is an aggressive malignant disease with limited therapeutic options. Current research is primarily focused on the relationship between copper and CRC. Innovative concepts, such as cuproplasia and cuproptosis, are being explored to understand copper‐related cellular proliferation and death. Cuproplasia is the regulation of cell proliferation that is mediated by both enzymatic and nonenzymatic copper‐modulated activities. Whereas, cuproptosis refers to cell death induced by excess copper via promoting the abnormal oligomerisation of lipoylated proteins within the tricarboxylic acid cycle, as well as by diminishing the levels of iron‐sulphur cluster proteins. A comprehensive understanding of copper‐related cellular proliferation and death mechanisms offers new avenues for CRC treatment. In this review, we summarise the evolving molecular mechanisms, ranging from abnormal intracellular copper concentrations to the copper‐related proteins that are being discovered, and discuss the role of copper in the pathogenesis, progression and potential therapies for CRC. Understanding the relationship between copper and CRC will help provide a comprehensive theoretical foundation for innovative treatment strategies in CRC management.

## Graphical abstract

- Physiological state copper ion transport process in vivo
- The role of copper ions in the carcinogenesis and development of colorectal cancer
- Copper ions mediate a variety of cell death modes
- The role of targeted copper ions in the diagnosis and treatment of colorectal cancer

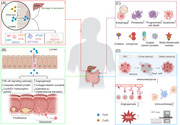

## 1 INTRODUCTION

Colorectal cancer (CRC) is the most common primary intestinal cancer and its incidence continues to increase. Currently, it is the third most frequent and second leading cause of cancer‐related mortality worldwide.^1^ CRC is characterised by multistage progression, often associated with poor prognosis, and frequently diagnosed in advanced stages.^2^ The proliferation and metastasis of CRC is multifactorial, including genetic predispositions, inflammatory bowel disease, dietary pattern with excessive meat intake and lifestyle factors.^3^ Recent studies have shown that copper ions are absorbed through the gastrointestinal tract and are present at elevated levels in CRC, mediating its carcinogenesis and development. Furthermore, copper ions can also trigger several types of cell death. Therefore, understanding the mechanism of action of copper ions in CRC and targeting them are crucial for the diagnosis and treatment of CRC.

A considerable number of studies have shown that copper, as a trace element in the human body, plays a vital role in various biological signalling pathways, which is mainly attributed to its redox‐active properties.^4^ The intricate copper homeostasis arises from the regulation of intracellular copper levels through a complex interplay of absorption, transport, storage and excretion mechanisms. Dysregulation of copper homeostasis has been associated with various diseases, particularly cancers.^5^, ^6^, ^7^, ^8^ The influence of copper on cancer cell characteristics is closely associated with cellular metabolism and antioxidant defence because many types of cancers require high levels of aerobic respiration and exhibit enhanced mitochondrial metabolism.^9^, ^10^

Copper can be absorbed from the daily diet, including copper‐rich foods such as offal and shellfish,^11^, ^12^ and is highly distributed in the liver, muscles, eyes and brain. Serum copper concentrations in healthy adults typically range from 70 mg/dL to 110 mg/dL.^13^ To maintain cellular health and avoid toxicity, the concentration of free copper in the cytoplasm varies from 10^−15 ^M to 10^−21 ^M.^14^ The intestinal tract is the centre of copper absorption and regulation, primarily mediated by Copper Transport Protein 1 (CTR1, Solute Carrier Family 31 member 1, SLC31A1, encoded by slc31a1) localised in the apical side of epithelial cells. After absorption through the gastrointestinal tract, copper ions are secreted into the bloodstream and bind to soluble chaperones such as ceruloplasmin, transcuprein, histidines, albumin and macroglobulins.^15^, ^16^, ^17^, ^18^ The gut is particularly vulnerable to copper imbalances because of its crucial role in copper absorption. Consequently, it is crucial to investigate the relationship between aberrant intestinal absorption of copper ions and the pathogenesis of CRC.

In this review article, we systematically summarise existing studies on the role of copper in CRC and describe the underlying mechanisms. First, the transport mechanisms of copper ions and their biological functions are reviewed in detail, highlighting diseases induced by aberrant copper ion levels and corresponding mechanisms. Furthermore, we describe the precise regulation of copper ion concentrations, which provides vital insights for clinical serological monitoring. Subsequently, this review elucidates in detail the role of copper ions in CRC proliferation and metastasis, and emphasises the importance of serum copper ions (mainly detected by plasma ceruloplasmin levels) in CRC surveillance, thus laying a solid foundation for basic cancer research. Considering that cuproptosis is mainly caused by copper overload, in this review, we also describe the mechanism of cuproptosis in the carcinogenesis and development of CRC, providing theoretical support for cuproptosis‐mediated malignant progression of CRC. Finally, we systematically explore various copper ion‐targeted treatment strategies for CRC, offering substantial theoretical support for therapeutic approaches. We hope that this review will deepen the understanding of this important metal ion, and thus facilitate the development of targeted therapies and biomarkers for clinical diagnostics.

## 2 THE PHYSIOLOGICAL HOMEOSTASIS AND METABOLISM OF COPPER

**FIGURE 1:**
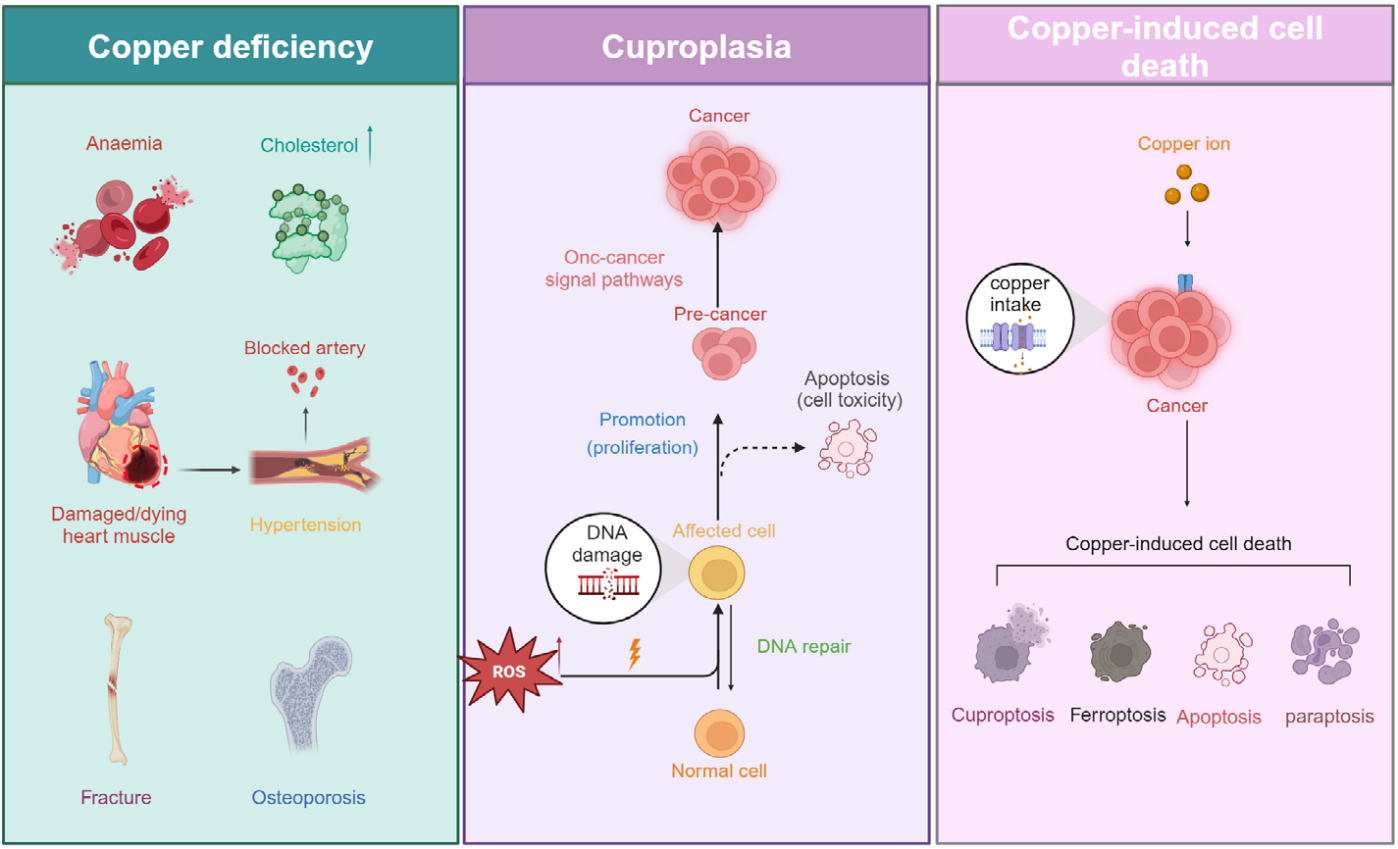
**Three roles of copper ion in the body**. Deficient copper levels can compromise biological systems, leading to disorders such as cardiovascular complications, anaemia, osteoporosis and Menkes disease. Conversely, elevated copper levels may facilitate tumour growth through the induction of ROS production, exacerbation of genomic instability and alteration of tumour‐associated signal transduction pathways. Additionally, when copper concentrations surpass a specific threshold, they can paradoxically induce tumour cell death. ROS, reactive oxygen species.

Copper, an essential transition metal, has an important bidirectional regulatory role with a U‐shaped dose response and is a dynamic cofactor for numerous enzymes, either donating or accepting electrons.^4^ Copper ions deficiency can impair biological systems, and may lead to various human diseases, including cardiovascular disorders,^19^ anaemia,^20^ osteoporosis^21^ and Menkes disease, a hereditary disorder caused by mutations in the ATPase copper transporting alpha (ATP7A) gene, which results in ATP7A deficiency.^22^, ^23^ Conversely, excessive copper accumulation can cause various metabolic disorders, ultimately leading to cell death.^24^ Preclinical studies have suggested that elevated copper levels promote cancer cell proliferation and progression both in vivo and in vitro.^5^, ^6^, ^25^, ^26^, ^27^, ^28^ This phenomenon, termed ‘cuproplasia’, may be associated with an increase in reactive oxygen species (ROS), and moderately elevated levels of copper can destabilise the genome and interfere with signalling pathways associated with cancer. However, copper overload can lead to severe health risks, including Wilson's disease,^29^ a rare genetic disorder caused primarily by mutations in the ATP7B gene, which is characterised by an excessive accumulation of copper in vital organs, leading to liver degeneration, organ damage and sclerosis.^30^ Previous studies have shown that disturbances in copper metabolism can lead to cancer cell death via apoptosis,^31^, ^32^ paraptosis,^33^, ^34^ ferroptosis^35^ and caspase independent cell death.^36^ In terms of molecular mechanisms, excessive copper ions can trigger cell death through multiple signalling pathways. Due to their redox‐active properties, copper ions participate in the Fenton reaction to produce ROS. This process leads to the release of cytochrome c and mitochondrial apoptosis‐inducing factor 1 into the cytoplasm, initiating caspase activation and DNA fragmentation.^31^, ^37^ Copper‐induced ROS also increase lipid peroxidation, deplete glutathione (GSH) and enhance cellular susceptibility to oxidative damage.^38^ Moreover, copper accumulation in the cell nucleus damages DNA in various biological systems and inactivates sulphur‐containing enzymes by binding to their sulphur groups.^39^ Additionally, copper ions inhibit the chymotrypsin‐like activity of the 20S proteasome and 19S proteasomal deubiquitinases, further inducing apoptosis.^40^ Research has also shown that excess copper induces paraptosis by inhibiting the ubiquitin‐proteasome system, triggering endoplasmic reticulum (ER) stress and caspase‐3 inhibition, leading to copper complex toxicity in HT‐1080 cells.^41^ Intracellular copper overload induces a noncaspase‐dependent form of paraptosis via ROS and an unfolded protein response.^42^ Concurrently, evidence indicates that copper plays dual role in promoting or inhibiting ferroptosis under specific conditions. Copper complexes, such as disulphiram/Cu^2+^ and elesclomol/Cu^2+^, disrupt mitochondrial homeostasis, leading to oxidative stress, thus inducing ferroptosis in hepatocellular carcinoma and CRC.^43^, ^44^ This is consistent with the observation that excessive copper accumulation produces a significant amount of ROS in cancer cells. Copper also promotes ferroptosis in pancreatic cancer by facilitating autophagic degradation of GPX4.^45^ Conversely, copper ions may also inhibit the occurrence of ferroptosis by limiting GPX4 expression in bathocuproinedisulphonic acid treatment.^46^, ^47^ Copper nanoparticles provide a feasible method of copper delivery by inducing ferroptosis in CRC cells.^48^ Therefore, ferroptosis is a metal‐dependent mode of cell death that involves both iron and copper. However, a recent study by Tsvetkov et al. demonstrated that copper‐dependent cell death is a unique form of cell death termed as cuproptosis.^49^ Cuproptosis is a recently identified cell death pathway that is triggered by the influx of excessive copper ions into cells. This pathway involves aberrant oligomerisation of copper‐dependent lipoylated proteins in the tricarboxylic acid (TCA) cycle, and a concurrent decrease in iron‐sulphur (Fe‐S) cluster proteins. Central to this process is Ferredoxin1 (FDX1), which plays a pivotal role in reducing Cu^2+^ ions to Cu^+^, facilitating dehydrolipoyltransacetylase (DLAT)‐mediated lipid acylation, and ultimately triggering cell death by decreasing the levels of Fe‐S cluster proteins. Inhibition of FDX1 or the enzymatic processes associated with lipid acylation halts cuproptosis, highlighting the significance of biomarkers such as FDX1, LIAS, LIPT1, DLD, DLAT, PDHA1, PDHB, MTF1, GLS and CDKN2A in the study and understanding of cuproptosis. Among these, the first seven genes mediate resistance to cuproptosis, whereas the latter three genes promote cuproptosis.^50^ These biomarkers are poised to become key indicators of this unique cell death mechanism.^49^, ^51^ Therefore, elucidating the mechanisms underlying copper‐induced cellular proliferation and death is essential for the advancement of anticancer therapies (Figure 1).

Herein, we summarise the major factors and signalling pathways involved in copper homeostasis, including cuproenzymes, membrane transporters and copper chaperones, which collectively regulate intracellular copper homeostasis. These proteins synergistically coordinate copper input, intracellular utilisation and output to maintain intracellular copper levels within a specific range. Therefore, we categorise the major regulators of copper metabolism according to their roles and highlight their essential functions in the modulation of this process (Table 1).

**TABLE 1:** Major regulators involved in copper homeostasis.

	Gene	Name	Subcellular location	Function	Refs
Copper absorption	CTR1	Copper transporter 1	PM	High‐affinity copper importer	17
	CTR2	Copper transporter 2	Organelle membrane	Regulation of CTR1	67, 68
	DMT1	Divalent metal transporter	PM	Copper uptake transporter, mainly complementary to CTR1 for copper uptake	62
	STEAP	Six‐transmembrane epithelial antigen of the prostate	PM	Metalloreductase, reduce Cu^2+^ to Cu^+^	56
	DCYTB	Duodenal Cytochrome b	PM	Metalloreductase, reduce Cu^2+^ to Cu^+^	54
Copper excretion	ATP7A	ATPase copper transporter 7A	Organelle Membrane, Regulated by the level of copper ions	Transport copper across cell membranes	6
	ATP7B	ATPase copper transporter 7B	Organelle membrane, regulated by the level of copper ions	Excrete copper into bile and plasma	22
Copper utilisation	ATOX1	Antioxidant protein	CP	Metallochaperone that delivers copper to ATP7A and ATP7B Cu^+^ transporters	92
	CP	Ceruloplasmin	Secreted	Major exchangeable plasma Cu carrier	16
	CCS	Copper chaperone for superoxide dismutase	CP	Metallochaperone that delivers copper to SOD	83
	SOD1	Superoxide dismutase 1	CP, Mit	Superoxide scavenger	84, 85, 86
	MT1/2	Metallothionein 1/2	CP	Bind divalent heavy metal ions, altering the intracellular concentration of heavy metals	82
	SCO1	Synthesis of cytochrome oxidase 1	Mit	Metallochaperone, required for copper delivery to the CuA site	127
	SCO2	Synthesis of cytochrome oxidase 2	Mit	Copper‐binding thiol‐disulphide oxidoreductase of COX2, a component of the copper delivery pathway to the CuA site	127
	COX11	Copper chaperone for cytochrome c oxidase 11	Mit	Metallochaperone, a component of the copper delivery pathway to the CuB site	104
	COX17	Copper chaperone for cytochrome c oxidase 17	Mit and CP	Metallochaperone that transfers copper to SCO1 and COX11 for cytochrome oxidase copper loading in mitochondria	87
	LOX	Lysyl oxidase	Secreted	Copper‐dependent enzymes, oxidise lysyl and hydroxylysyl residues in collagen and elastin	97

Abbreviations: CP, cytoplasm; Mit, mitochondria; NC, nucleus; PM, plasma membrane.

### 2.1 Copper absorption and excretion

Copper is absorbed via the intestinal tract, mainly in the small intestine, especially in the proximal part of the small intestine, including the duodenum and jejunum.^52^ Copper ions exist in two valence states, Cu^+^ and Cu^2+^, with Cu^+^ being predominant in the reducing intracellular environment. Physiologically, CTR1 primarily facilitates the transport of monovalent copper ions into the intestinal epithelium. Consequently, metalloreductases such as the Six‐Transmembrane Epithelial Antigen of the Prostate (STEAP) and duodenal cytochrome b act as copper reductases,^53^, ^54^, ^55^, ^56^ converting divalent copper into monovalent copper, thereby enhancing the uptake of copper ions by CTR1. The STEAP family of proteins consists of four members, namely, STEAP 1−4, whose primary role is to promote metal homeostasis by reducing the oxidation states of iron and copper, thereby facilitating the absorption of these metals.^57^, ^58^ However, STEAP1 is an exception in that it does not exhibit metalloreductase activity, probably because of the lack of the F420H2:NADP+ oxidoreductase structural domain and Rossmann folded structure.^59^ Furthermore, copper is also transported via divalent metal transporter 1 to a lesser extent.^60^, ^61^, ^62^, ^63^ Copper is then transported to the opposite side of the epithelium through the copper chaperone antioxidant 1 (ATOX1) and subsequently released into the bloodstream via ATP7A.^64^

Accumulating evidence indicates that CTR1 is indispensable for copper transport to specific organs and that its regulation is copper‐dependent.^65^ Studies by Nose et al. and Lee et al. established that CTR1 plays a crucial role in copper ion uptake in intestinal‐specific and systemic knockouts, respectively. Notably, mice with systemic Ctr1 knockout (Ctr1 − / −) experienced uterine death during the second trimester.^66^, ^67^ Additionally, CTR2 acts as a copper sensor within endosomal compartments.^68^, ^69^ Studies including those by Helena et al. have shown that CTR2 knockout in mice leads to progressive age‐related copper hyperaccumulation, especially in the brain.^68^ Therefore, investigating CTR2‐dependent cleavage of CTR1 is crucial for understanding the regulation of copper absorption in various tissues and under different physiological or pathological conditions.

**FIGURE 2:**
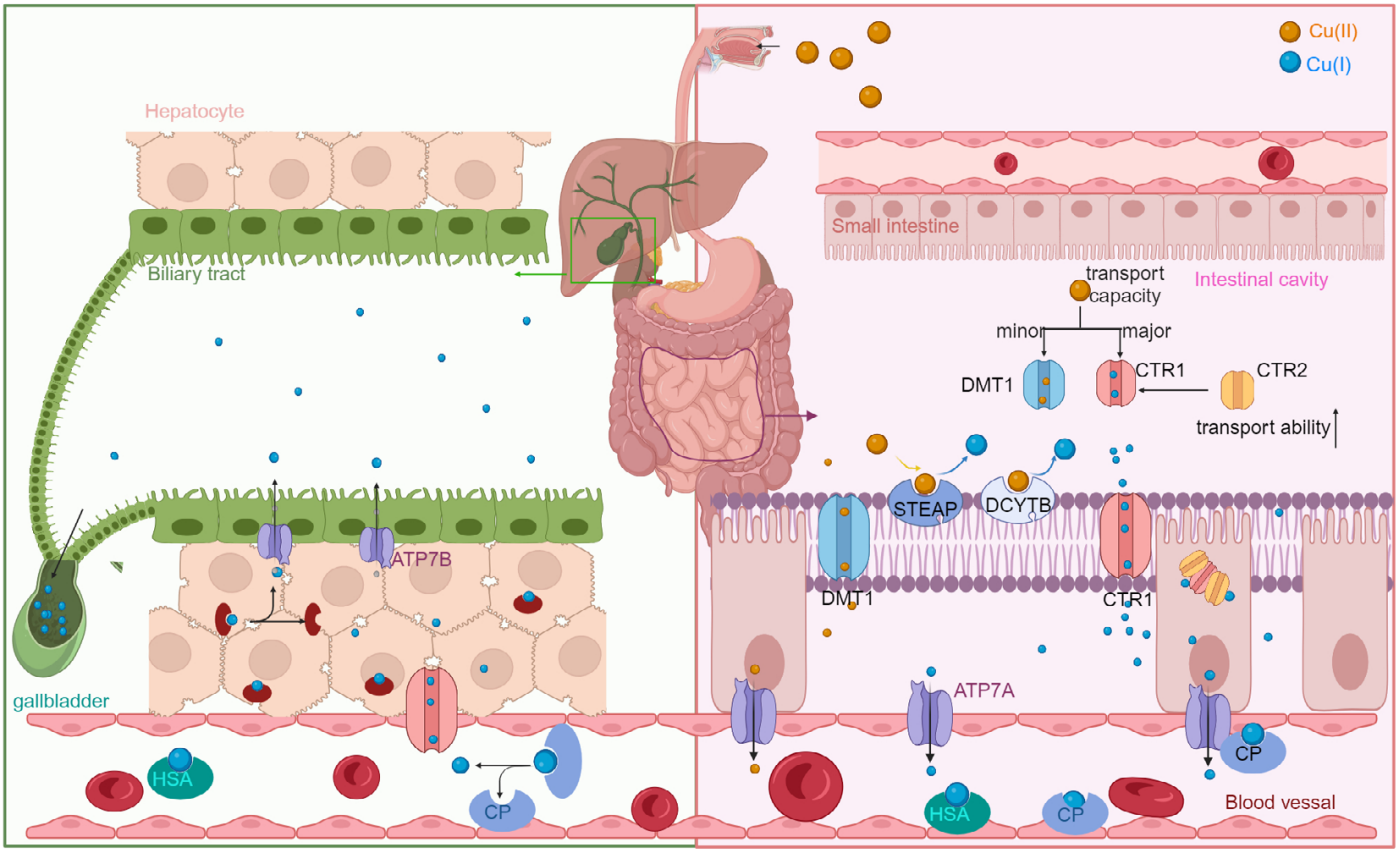
**The schematic illustrates the systemic and cellular metabolism of copper**. Copper is absorbed primarily through the small intestine and then transported via the bloodstream to the liver for storage and subsequent excretion into the bile. At the cellular level, copper uptake occurs predominantly through CTR1, and to a lesser extent DMT1. CTR1 is chiefly responsible for transporting monovalent copper ions, while STEAP and DCYTB are copper reductases. In the bloodstream, copper ions are primarily bound to CP and HSA. In the biliary system, copper ions are mainly excreted through ATP7B. STEAP, Six‐Transmembrane Epithelial Antigen of the Prostate; DCYTB, Duodenal Cytochrome b; CTR1/2, Copper Transport Protein 1 and 2; DMT1, divalent metal transporter 1; ATP7A and ATP7B, ATPase copper transporter 7A and 7B; CP, ceruloplasmin; HSA, human serum albumin.

In addition to copper absorption, copper export plays a crucial role in preventing copper accumulation and cytotoxicity. The gallbladder is the main organ involved in copper excretion, and small amounts of copper are eliminated from the body through sweat.^70^, ^71^, ^72^ The core molecular mechanisms of this process involves Cu‐ATP7A and ATP7B, whose localisation and activity are heavily influenced by copper levels. Under physiological conditions, these transporters are located in the trans‐Golgi network (TGN) where they transfer copper ions from the cytoplasm to the TGN lumen. When intracellular copper concentrations are elevated to toxic levels, ATP7A and ATP7B translocate from the TGN to the vesicular region and merge with the plasma membrane, thereby promoting copper export.^73^ Moreover, ATP7A and ATP7B exhibit distinct expression patterns. ATP7A is ubiquitously expressed in almost all cell types, except hepatocytes. In contrast, ATP7B is predominantly expressed in the liver^74^ and plays a key role in the transport of copper ions into the bloodstream, especially during excretion through the liver and gallbladder. These mechanisms cumulatively contribute to the harmonious and stable balance of copper in the body, including intestinal absorption, hepatic storage and biliary excretion (Figure 2).

### 2.2 Copper utilisation

To avoid cytotoxicity and ensure precise copper homeostasis, copper ions in the blood are predominantly bound to proteins. Approximately 75% of nonexchangeable copper ions bind to ceruloplasmin (CP), and approximately about 25% of exchangeable copper ions bind to human serum albumin (HSA). Only a marginal 0.2% of copper ions binds to histidine.^75^, ^76^

**FIGURE 3:**
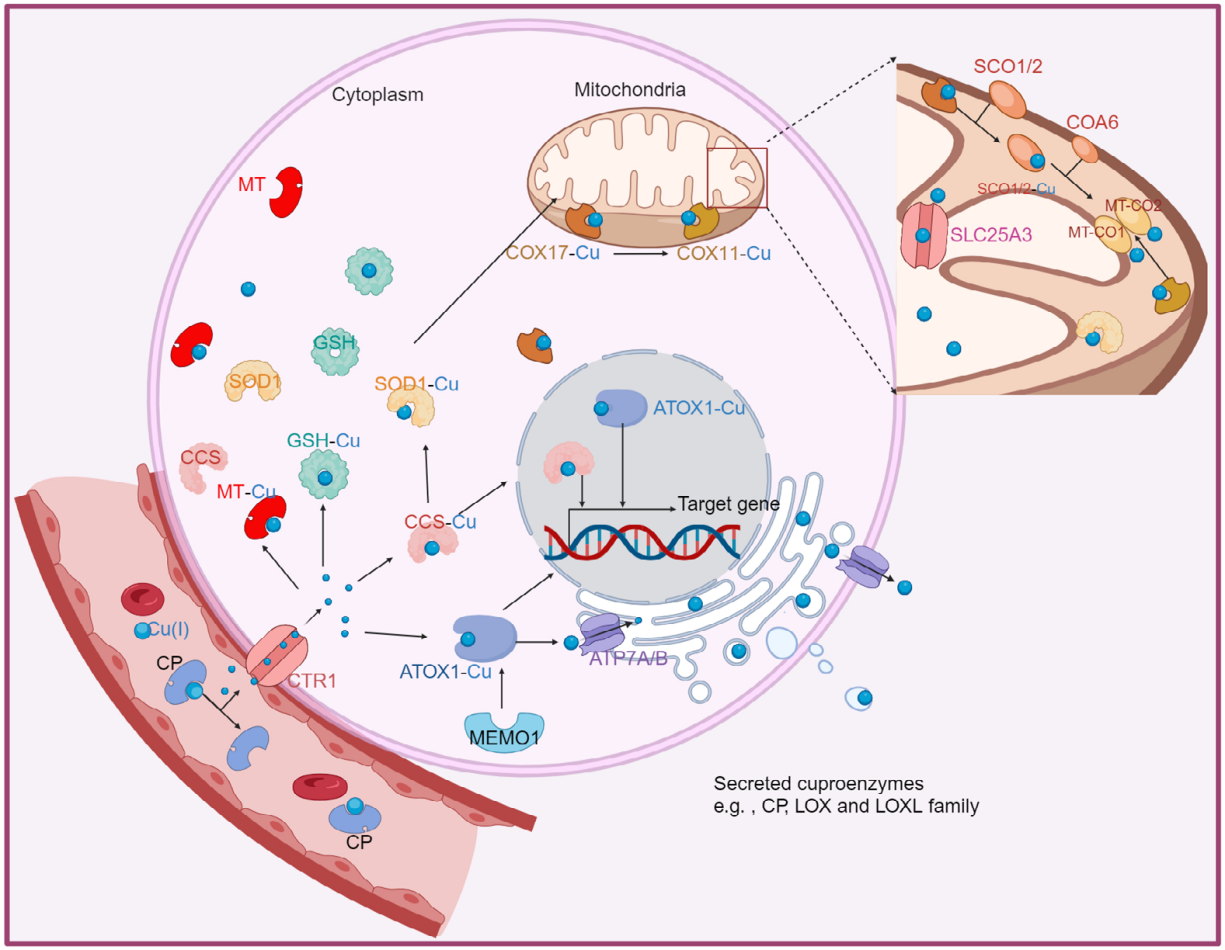
**The key molecular mechanisms of copper metabolism**. Extracellular Cu^2+^ are reduced to Cu^+^ by the reductase STEAP and then imported into the cell via the CTR1. Inside the cell, Cu^+^ is distributed to cytosolic copper chaperones, including MT, GSH, CCS and SOD1, which in turn deliver it to specific subcellular locations such as the mitochondria, TGN and nucleus. CP, ceruloplasmin; CTR1, Copper Transport Protein 1; MT, Metallothionein; GSH, glutathione; SOD1, superoxide dismutase 1; CCS, copper chaperone for superoxide dismutase; ATOX1, antioxidant protein; MEMO1, mediator of cell motility 1; ATP7A and ATP7B, ATPase copper transporter 7A and 7B, respectively; TGN, trans‐Golgi network; CCO, cytochrome c oxidase; COX17, cytochrome c oxidase copper chaperone17; COX11, cytochrome c oxidase copper chaperone 11; SLC25A3, solute carrier family 25 member 3; SCO1/2, synthesis of cytochrome c oxidase 1 and 2; COA6, cytochrome c oxidase assembly factor 6; MT‐CO1/2, mitochondrially encoded cytochrome c oxidase 1 and 2; LOX, lysyl oxidase; LOXL, lysyl oxidase like.

Under physiological conditions, copper is transported to various cellular substructures including the Golgi apparatus, cytoplasm, mitochondria, and nucleus (Figure 3). The cytoplasm contains high concentrations of GSH and metallothionein (MT), natural chelators of copper ions.^77^, ^78^ These proteins, which greatly outnumber copper ions, have two main functions: (1) maintaining a negative concentration gradient across the cytoplasmic membrane, thereby promoting CTR1‐mediated copper uptake^79^; and (2) acting as cytoplasmic copper buffers, binding free copper ions to prevent cytotoxicity, and producing ROS.^6^, ^80^, ^81^, ^82^ MT contains metal–thiolate clusters that bind heavy metals and can chelate large amounts of copper ions. Interestingly, increased intracellular copper ion levels also stimulate MT expression.^83^ Consequently, MT and GSH form intrinsic defence mechanisms against copper‐induced cytotoxicity. Additionally, copper ions can bind to chaperones, especially the copper chaperonin of superoxide dismutase (CCS) which interacts with copper ions and transports them to superoxide dismutase 1 (SOD1), facilitating the formation of disulphide bonds that are essential for its proper structure and enzymatic activity.^84^, ^85^ Furthermore, CCS regulates SOD1 distribution in the intermembrane space and cytoplasm in an oxygen‐dependent manner. This regulatory mechanism is essential for maintaining ROS levels in vivo and mitigating ROS generated by the electron transport chain, thereby preventing oxidative damage caused by copper overloading.^86^, ^87^

Furthermore, copper ion chaperones in the mitochondria play a crucial role in the function of cytochrome c oxidase (COX), a key component of oxidative phosphorylation. The entry of copper ions into the mitochondria is mainly dependent on COX17,^88^ which transports Cu^+^ from the cytoplasm to mitochondrial membrane proteins, such as synthesis cytochrome C oxidase 1 and cytochrome C oxidase 2,^89^ facilitating copper insertion into mitochondrial encoded cytochrome c oxidase II (MT‐CO2/COX2).^90^ COX17 also transports copper to mitochondria‐encoded cytochrome c oxidase I (MT‐CO1/COX1) via the cytochrome c oxidase copper chaperone 11 (COX11),^90^ which drives the electrochemical production of ATP.^91^ Therefore, the cellular copper pool is closely associated with mitochondrial oxidative phosphorylation, in which COX17 is essential for MT‐CO1 and MT‐CO2 activity in the mitochondrial respiratory chain.^92^

Copper is transported to the nucleus by the CCS and ATOX1 proteins. Here, it initiates the activation of transcription factors such as hypoxia‐inducible factor 1 via the CCS^26^ and acts as a copper‐dependent transcription factor via ATOX1.^93^ Additionally, the copper chaperone ATOX1 transports copper to the trans‐Golgi network and delivers it to the copper‐dependent ATPases, ATP7A and ATP7B.^94^ MEMO1 (mediator of cell motility 1) also plays a crucial role in enhancing Cu(I) binding to ATOX1, thereby alleviating the excess of Cu‐induced ROS production.^95^ Moreover, copper metalloenzymes are essential for several critical cellular biological processes. These include promoting pigmentation via tyrosinase,^96^ influencing neural signalling via dopamine β‐hydroxylase,^97^ facilitating the cross‐linking of elastin and collagen primarily via lysyl oxidase (LOX) and lysyl oxidase‐like 2 (LOXL2),^98^ and aiding leukocyte trafficking via amine‐containing oxidase copper 3 (AOC3).^99^ Copper is also an important component of the myelin sheath that protects neurons.^61^ Taken together, these are the main mechanisms by which copper ions function in the human body.

## 3 ROLE OF COPPER AND CUPROPTOSIS IN CRC

### 3.1 Copper and carcinogenesis in CRC

Elevated copper ion levels have been associated with tumourigenesis and malignant progression, both in vivo and in vitro.^5^, ^6^, ^27^ This phenomenon is known as ‘cuproplasia’ and refers to copper‐dependent cell growth, including enzymatic and nonenzymatic activities regulated by copper.^27^ Specifically, copper ions function as allosteric modulators, activating the RAF‐MEK‐ERK signalling pathway by binding to specific enzymes such as MEK1 and MEK2, thereby improving their capacity to phosphorylate ERK1 and ERK2.^100^, ^101^, ^102^, ^103^ This activation promotes cancer cell proliferation and growth. Additionally, copper ions stimulate the activity of the E2‐binding enzymes UBE2D1‐UBE2D4 through positive allosteric activation, thereby facilitating protein degradation.^104^ In the context of cancer, this mechanism may lead to ES becoming a prominent copper ionophore in cancer therapy. Its primary function is to induce oxidative stress ion and subsequent degradation of key oncogenic proteins, including p53, affecting cell cycle regulation, inhibiting programmed cell death, and thereby enhancing the viability of malignant cells. Recent studies have found higher copper levels in CRC tissues and patient sera (mainly detected by plasma CP levels) than in healthy individuals,^105^, ^106^, ^107^ indicating that copper plays an important role in CRC development and cell proliferation. Notably, the serum copper/zinc ratio increased progressively with the advancing stages of CRC progression.^107^ Strikingly, elevated serum copper levels are strongly correlated with the degree of malignancy in CRC.^108^ A significant increase in the expression of the copper translocation regulator, ATP7A, was observed in KRAS mutation‐specific CRC cells. This increase facilitates a high influx of copper into the cells, supporting CRC proliferation.^109^ This indicates that copper signalling may play a crucial role in CRC development. Specifically, copper ions modulate signalling pathways associated with nutrient sensing by positively regulating the activities of two kinases, ULK1 and ULK2, and this regulation affects cell growth and proliferation and supports the survival of cancer cells.^7^, ^110^ Inflammatory cytokines, such as interleukin (IL)−17, have been shown to induce STEAP4‐dependent cytosolic copper uptake. This uptake activates the E3 ligase XIAP, which in turn enhances IL‐17‐induced nuclear factor NF‐kappaB (NF‐κB) activation and inhibits caspase 3 activity, thereby promoting intestinal tumourigenesis in mice.^111^

### 3.2 Copper and metastasis in CRC

**FIGURE 4:**
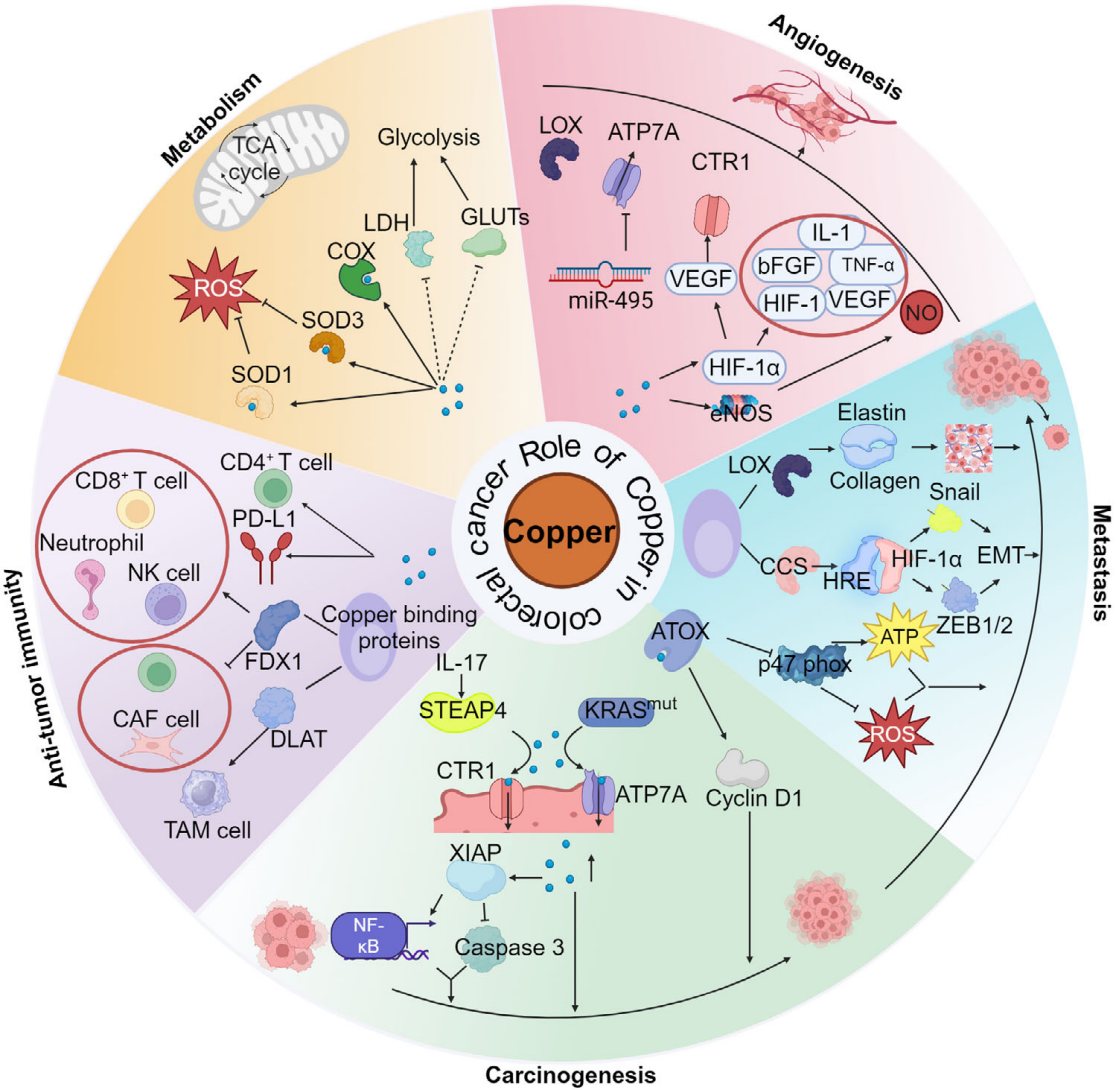
**Increased copper levels and copper‐binding proteins promote CRC proliferation and metastasis**. In CRC proliferation, three key mechanisms are involved. First, the proliferation of CRC cells is associated with increased levels of ATOX1. Second, an increase in copper influx into cancer cells further promotes CRC proliferation. Finally, the inflammatory cytokine IL‐17 elevates intracellular copper levels by stimulating STEAP4 expression. In the context of CRC metastasis, first, ATOX1 facilitates this process by reducing p47phox expression, which in turn decreases ROS production and enhances ATP synthesis. Second, it significantly influences the EMT process. Third, copper is vital in angiogenesis. It binds to and modulates proteins essential to angiogenic processes. ATOX1, Antioxidant‐protein 1; STEAP4, Six‐Transmembrane Epithelial Antigen of the Prostate 4; ROS, reactive oxygen species; XIAP, X‐linked inhibitor of apoptosis; CTR1, Copper Transport Protein 1; ATP7A, ATPase copper transporter 7A; eNOS, endothelial nitric oxide synthase; NO, nitric oxide; HIF1, hypoxia inducible factor 1; CP, ceruloplasmin; VEGF, vascular endothelial growth factor; CCS, copper chaperone for SOD1; HRE, hypoxia response element; ZEB1/2, zinc finger E‐box binding homeobox 1 and 2; Snail, snail family transcriptional repressor 1; LOX, lysyl oxidase; EMT, epithelial‐to‐mesenchymal transition; ECM, extracellular matrix.

Elevated copper levels are critical not only for the development of CRC but also for its metastasis, particularly to the liver, which is the most common site of distant CRC metastasis and a storage organ for copper.^14^ Recent studies have shown that copper levels in CRC liver metastases are significantly higher than those in normal liver tissues.^112^ Additionally, copper and copper‐binding proteins are vital for epithelial‐mesenchymal transition (EMT) and angiogenesis in CRC metastasis. Copper is essential for LOX and LOXL proteins, which are involved in collagen and elastin crosslinking. LOX secretion by cancer cells remodels the extracellular matrix to form premetastatic niches, thereby recruiting bone marrow‐derived cells, and promoting EMT in CRC.^113^ Moreover, copper‐mediated interactions between hypoxia response elements and HIF‐1α activate the transcription factors ZEB1, ZEB2 and Snail via CCS, thereby promoting EMT in CRC.^26^, ^98^, ^114^ In addition to copper ions and copper‐binding proteins, ATOX1, a key molecule that maintains copper homeostasis, plays a significant role in CRC proliferation and metastasis. ATOX1 also serves as a transcription factor that regulates the expression of Cyclin D1 and promotes cell proliferation by binding to copper.^93^ Additionally, ATOX1 is markedly overexpressed in metastatic CRC tissues and cell lines, with a significant increase in its nuclear localisation. The entry of ATOX1 into the nucleus can activate transcription and promote the expression of CyclinD1 and the NADPH oxidase subunit p47 phox. Notably, this effect disappeared when the copper‐binding structural domain of ATOX1 is knocked out, suggesting that ATOX1‐mediated CRC metastasis is copper‐dependent.^115^ In summary, copper and copper‐binding proteins are inextricably linked to CRC proliferation and metastasis (Figure 4).

### 3.3 Copper, angiogenesis and cell metabolism in CRC

Angiogenesis, the process of creating new capillaries from preexisting vasculature, involves an intricate sequence of events that includes endothelial cell proliferation, migration, differentiation and extracellular matrix remodelling. This process is crucial for normal physiological functions and certain pathological conditions such as malignant cancers.^116^ Research indicates that cancer cells exhibit an angiogenic phenotype marked by the presence of numerous angiogenic molecules. This signifies a disturbance in the equilibrium of endogenous anti‐angiogenic factors, thereby instigating cancer‐associated angiogenesis.^117^ In this progression, copper ions play a critical role in the early stages of cancer vasculature formation by stimulating endothelial cell proliferation and migration. Angiogenic factors that have been identified to be activated by copper include basic fibroblast growth factor, vascular endothelial growth factor (VEGF), tumour necrosis factor‐alpha and IL‐1, which bind to endothelial cells, facilitating their transition from the G0 phase to the G1 phase and promoting proliferation. Quintin et al. demonstrated that a copper‐deficient diet or the use of tetrathiomolybdate (a copper chelator) leaded to a reduction in copper levels, which impeded angiogenesis by suppressing the expression of the aforementioned angiogenic factors and inducing endothelial cell regression to the G0 phase or apoptosis.^118^ Furthermore, copper is essential for the activation of HIF‐1, a key transcription factor regulating VEGF expression. The presence of copper ensures the formation of an HIF‐1 transcription complex, which activates the expression of target genes, including VEGF. However, excess copper can lead to the accumulation of the rate‐limiting component of HIF‐1, HIF‐1α, in the cytoplasm, thereby activating HIF‐1.^119^ Furthermore, copper ions mediate the activity of endothelial nitric oxide synthase and increase the production of the vasodilator nitric oxide, thereby promoting angiogenesis.^120^

The precise mechanism through which copper stimulates angiogenesis in CRC remains unclear. Most studies have focused on other forms of cancer, in which copper activates numerous angiogenic factors. For example, Damiano et al. observed that copper ions, through the activation of the EGFR/ERK/c‐fos signalling pathway, induced the expression of G protein‐coupled oestrogen receptor 1, HIF‐1α, and VEGF in breast and liver cancer cells, thereby promoting tumour angiogenesis.^121^ In patients with malignant ovarian cancer, increased copper level has been observed, suggesting that the angiogenic effects of copper in ovarian cancer and endothelial cells might influence the production of ascites. Therefore, strategies to reduce copper levels in ascites may be beneficial for downregulating VEGF expression and improving the prognosis of malignant ovarian tumours.^122^ Genes associated with copper ion transport, including CTR1, ATP7A and LOX, also promote angiogenesis. Archita et al. showed that CTR1 acts as a redox sensor that enhances angiogenesis in endothelial cells. The absence of CTR1 in cardiovascular cells weakens the response to VEGF‐induced VEGFR2 signalling and angiogenesis. Mechanistically, upon VEGF stimulation, the cytoplasmic C‐terminal Cys189 of CTR1 undergoes rapid sulphenylation, inducing the formation of a CTR1‐VEGFR2 disulphide bond and co‐internalisation into early endosomes, thereby sustaining VEGFR2 signalling.^123^ MiR‐495 combats cisplatin resistance and inhibits angiogenesis in oesophageal cancer cells by targeting ATP7A expression.^124^ LOX overexpression and copper‐induced LOX activity promote angiogenesis and proliferation in oral squamous cell carcinoma cells.^125^

In terms of cellular metabolism, copper is an essential cofactor for many enzymes.^126^ In cancer cells, changes in copper ion levels can prompt a metabolic shift from mitochondria‐dependent oxidative phosphorylation to a more glycolysis‐dependent state, a phenomenon known as the ‘Warburg effect’. Metabolic reprogramming is associated with the regulation of the activities of many key enzymes by copper ions.^101^, ^127^ These include cytochrome c oxidase (COX, also known as Complex IV), a crucial enzyme complex in the mitochondrial electron transport chain that directly uses copper ions as cofactors.^91^, ^128^ SOD, especially SOD1 (cytoplasmic) and SOD3 (extracellular), use copper and zinc as cofactors. SOD is essential for regulating intracellular ROS levels. Changes in copper ions can affect SOD activity, thereby influencing the cellular response and regulation of ROS, which plays an important role in regulating the metabolic reprogramming of cancer cells. Other enzymes include the ubiquitin ligase system, lactate dehydrogenase (LDH) and glucose transport proteins. For example, studies have reported high expression of COX in CRC,^129^, ^130^, ^131^ where COX subunit 1 plays a prognostic role in distinguishing between CRC and adenoma.^132^ Sabrina et al. reported the involvement of the mitochondrial copper chaperone protein COX19 in the biosynthesis of human COX.^133^ Furthermore, studies have indicated that the suppression of COX19 expression in nonsmall cell lung cancer cells could enhance apoptosis.^134^ Moreover, Gao et al. reported that COX19 was also associated with the prognosis of patients with CRC, primarily through MACC1 transcriptional upregulation of COX19, and COX19 promoting intracellular copper transport and enhancing mitochondrial activity to exert its carcinogenic effects.^135^, ^136^

### 3.4 Copper and the antitumour immune response in CRC

In the 1950s, researchers discovered that deficiency of copper led to hypocupremia, hypoceruloplasminemia and neutropenia in infants, which subsequently affected the ability of their immune system to kill bacteria.^137^, ^138^, ^139^ The influence of copper on the immune system has garnered growing research interest following these findings. In 2009, studies by White et al. further revealed that the effect of copper ions on macrophages was closely associated with the expression of ATP7A, and proinflammatory factors such as LPS or IFN‐γ could enhance the absorption of copper by macrophages, increasing the expression of CTR1 and ATP7A, thereby augmenting their pathogen‐killing capabilities.^140^, ^141^ Moreover, copper deficiency was found to affect acquired immunity, as evidenced by the weakened response of cytotoxic T lymphocytes in mice against allogeneic antigens, highlighting the critical role of copper in immune function regulation.^142^

Concurrently, emerging research has reported relationship between tumour immunity and copper, such as the association of reduced copper with CD4+ T cell infiltration in mouse mesothelioma^143^ and the influence of the antitumour drug Dp44mT on T cell activity through a copper‐related mechanism.^144^ Regarding CRC immune response, bioinformatics studies constructing a copper‐related death prognostic index (CPS score) have indicated a significant positive correlation between CPS scores and both microsatellite instability high (MSI‐H) and tumour mutation burden (TMB), suggesting that patients with higher CPS scores possess higher immunogenicity, thereby potentially benefiting more from immune checkpoint blockade therapy.^145^, ^146^ By constructing a prognostic risk model utilising copper‐associated long noncoding RNAs (lncRNAs; CRLncSig), it was uncovered that the low‐risk group demonstrated prolonged overall survival (OS) in comparison to the high‐risk group. Additionally, the low‐risk group exhibited decreased tumour purity, enhanced immune cell infiltration, elevated immune scores, enriched immune activation pathways, and a more favourable response to immunotherapy.^147^ The role of cuproptosis in CRC immunotherapy has primarily focused on FDX1 and DLAT. Analysis using the TCGA database showed that FDX1 is positively correlated with immune cell infiltration in colon adenocarcinoma, particularly indicating a higher proportion of CD8+ T cells in tumour tissues compared to adjacent nontumour tissues, while the proportion of CD4+ T cells was opposite, suggesting that FDX1 is a potential target for colon adenocarcinoma immune therapy.^148^ DLAT is significantly associated with immune‐related pathways in pan‐cancer, liver cancer, and CRC. Moreover, DLAT expression correlated with tumour‐associated macrophage infiltration, TMB and MSI. These studies indicate that DLAT plays a crucial role in cancer immunity and development, potentially serving as a prognostic biomarker for cancer immune therapy.^149^, ^150^ Furthermore, DLAT promotes antitumour immune activation by reversing T cell exhaustion and inducing apoptosis.^151^

### 3.5 Role of cuproptosis in CRC

Few studies have addressed the molecular mechanisms of cuproptosis in CRC. In other digestive system cancers, such as in gastric cancer, copper stress can promote the site K229 lactylation of METTL16, which in turn mediates m6A‐modification on FDX1 mRNA, thereby promoting cuproptosis.^152^ Furthermore, ferroptosis inducers sorafenib and erastin promote cuproptosis in primary liver cancer by suppressing the degradation of FDX1 protein and reducing synthesis of intracellular copper chelator GSH through the suppression of cystine transportation.^153^ These results reveal the significance of cuproptosis induction as a promising therapeutic strategy for cancer.

Furthermore, the application of multiomics analysis has expanded our understanding of the role of cuproptosis in CRC. Research has indicated that by analysing cuproptosis‐related genes (CRGs), it is possible to classify CRC patients into different risk groups and identify different molecular subtypes with different clinical characteristics. For instance, one study categorised patients with CRC into two molecular subtypes based on 41 CRGs, with subtype C1 characterised by lower OS rates and advanced clinical staging.^154^ Moreover, cuproptosis is closely linked to the tumour immune microenvironment. Patients classified in the low‐risk group exhibiting stronger immunogenicity, higher immune scores, and a greater degree of microsatellite instability, suggesting a better response to immunotherapy.^50^, ^145^, ^155^, ^156^, ^157^, ^158^, ^159^ Further investigation have highlighted the potential role of cuproptosis‐associated microRNAs and lncRNAs in CRC. For example, six cuproptosis‐associated miRNAs including hsa‐miR‐653, hsa‐miR‐216a, hsa‐miR‐3684, hsa‐miR‐4437, hsa‐miR‐641 and hsa‐miR‐552 were used to construct prognostic models. It was found that miR‐653 was overexpressed in CRC tissues, promoting cell proliferation and inhibiting apoptosis by negatively regulating DLD expression.^160^ Similarly, certain lncRNA combinations have demonstrated potential for predicting CRC prognosis, including SNHG16, LENG8‐AS1, LINC0225, and RPARP‐AS1.^118^ These molecular markers not only provide new biological insights but also offer potential targets for future targeted therapy strategies. In terms of drug sensitivity, a study discovered that CRC patients with different risk scores had different sensitivities to specific drugs, such as greater sensitivity to AKT inhibitors and doxorubicin in the high‐risk group, which lays the groundwork for personalised treatment.^154^ Elesclomol‐copper rapidly inhibited the growth of CRC cells and oxaliplatin‐resistant cell lines, with 4‐octyl itaconate (4‐OI) inhibiting aerobic glycolysis by targeting GAPDH, thereby promoting cuproptosis.^161^ Furthermore, CRC cells treated with elesclomol‐copper showed increased E2F3 expression, which significantly enhanced the resistance of colorectal adenocarcinoma (COAD) cells to elesclomol.^162^

Multiomics analysis has also revealed the role of known molecular markers associated with cuproptosis in CRC progression. For example, low expression of FDX1 in COAD indicated poor prognosis, and immune microenvironment analysis showed that the proportion of CD8+ T cells was significantly lower than that of neighbouring normal tissues, whereas the proportion of CD4+ T cells showed the opposite trend.^148^ Additionally, FDX1 inhibited CRC growth and progression by suppressing EMT.^163^ Single‐cell and bulk RNA sequencing analyses identified AOC3, COX11, COX17, COX19, CCS, CDKN2A, DLD, DLAT, and PDHB as CRGs predictive of CRC prognosis, revealing that the elevated expression of COX17 in CD4‐CXCL13 T cells mediated Treg infiltration and T cell exhaustion, while DLAT reversed T cell exhaustion and induced apoptosis, promoting antitumour immune activation.^151^ Moreover, multiomics analysis identified the novel cuproptosis core‐related gene TIGD1, which significantly regulates CRC cuproptosis, and offers new targets for CRC treatment.^164^

In summary, as a novel mechanism of cell death, cuproptosis has extensive potential applications in CRC research, encompassing areas ranging from prognostic assessment and molecular typing to the development of therapeutic strategies. A comprehensive analysis of the roles of cuproplasia and cuproptosis in CRC will provide a new perspective on the complexity of CRC. Such studies will also help identify new targets and strategies for future CRC treatment.

## 4 ROLE OF COPPER IN THE TREATMENT AND DIAGNOSIS OF CRC

Elevated copper levels in CRC compared with adjacent normal tissues, coupled with growing evidence that high copper levels facilitate CRC proliferation and metastasis, make targeting copper ions a promising treatment for CRC. Currently, cancer therapeutic agents involving copper primarily consist of copper chelators, copper ionophores, copper‐containing compounds and the radioisotope ^64^Cu.^114^, ^165^ We categorised and detailed the roles and mechanisms of these agents in the diagnosis and treatment of CRC (Table 2). Additionally, we systematically organised and summarised the clinical trial data pertaining to these drugs, particularly the more established copper‐targeting cytotoxic agents such as tetrathiomolybdate (TM) and disulphiram (DSF) – in cancer therapy (Table 3).

**TABLE 2:** Mechanisms of different types of agents related to copper for CRC treatment.

	Name	Mechanisms	Result	Refs
Copper chelators	TM	Regulated phosphorylation levels of ERK1/2; increased expression of hCTR1 protein	Inhibited CRC proliferation, survival and migration; increased oxaliplatin sensitivity	117, 167
	Trientine	Inhibited the angiogenic factors, particularly VEGF and IL8	increased CRC apoptosis	172
	TPEN	Engaged in redox cycling to generate hydroxyl radicals; increased ROS accumulation and Mcl‐1 ubiquitination	increased CRC apoptosis; overcame drug resistance	170
	JYFY‐001	Decreased CRC extracellular acidification rate and oxygen consumption rate	Inhibited CRC proliferation; Increased CRC apoptosis	175
	D‐penicillamine	Increased expression of hCTR1 protein	increased oxaliplatin sensitivity	169
	Bathocuproinedisulphonic acid	Increased expression of hCTR1 protein	increased oxaliplatin sensitivity	166
Copper ionophores	ES	promoted the degradation of ATP7A; increased ROS accumulation and promoted SLC7A11 degradation	promoted CRC ferroptosis	178, 179
	DSF	miR‐16‐5p and 15b‐5p/ALDH1A3/PKM2 axis‐mediated aerobic glycolysis pathway; upregulated ULK1; induced molecules expression of cell ICD	inhibited CRC progression; Induced autophagic; increased apoptosis	185, 186, 187, 188, 189, 190, 191, 192, 193
Copper‐containing compounds	copper(II) complex	Activated caspases 3/7/9, BAX and ROS accumulation	Increased apoptosis	211
	copper(I) complex	Activated p53 pathway; suppressed the ubiquitin‐proteasome pathway and triggered endoplasmic reticulum stress; increased ROS accumulation	Increased apoptosis	210
	Copper nanoparticles	Triggered cell autophagy‐dependent apoptosis and ferroptosis; generated cancericidal species ·OH and 1O2 from rich H2O2 in tumours for nanocatalytic tumour therapy; reprogram the macrophages from the M2 phenotype to the M1 phenotype	Inhibited CRC proliferation and metastasis; increased apoptosis and autophagy	225, 226, 227, 228, 229

**TABLE 3:** Clinical trials evaluating agents that target copper for the treatment of cancer.

	Name	Cancer types	NCT Number	Phases	Enrolment	Status
Copper chelators	TM	CRC	NCT00176774	II	24	Completed
		Nonsmall cell lung cancer	NCT01837329	I	26	Completed
		Oesophageal carcinoma	NCT00176800	II	69	Completed
		Prostate cancer	NCT00150995	II	19	Completed
	Trientine	Advanced malignancies	NCT01178112	I	56	Completed
		Ovarian cancer	NCT03480750	I/II	18	Completed
	D‐penicillamine	Glioblastoma	NCT00003751	II	40	Completed
Copper ionophores	ES	Melanoma	NCT00522834	III	630	Terminated
		Melanoma	NCT00084214	I/II	103	Completed
		Prostate cancer	NCT00808418	I	34	Completed
		Soft tissue sarcomas	NCT00087997	II	80	Completed
		Ovarian cancer, fallopian tube cancer, or primary peritoneal cancer	NCT00888615	II	58	Completed
		Acute myeloid leukaemia	NCT01280786	I	36	Active
		Solid tumours	NCT00827203	I	30	Suspended
	DSF	Breast cancer	NCT03323346	II	150	Recruiting
		Gastric cancer	NCT05667415	N/A	40	Not yet recruiting
		Glioblastoma	NCT02678975	II/III	88	Completed
		Nonsmall cell lung cancer	NCT00312819	II/III	60	Completed
		Prostate cancer	NCT01118741	N/A	19	Completed
		Prostate cancer	NCT02963051	I	9	Terminated
		Pancreas cancer	NCT03714555	II	1	Completed
		Refractory solid tumours involving the liver	NCT00742911	I	21	Completed
		Melanoma	NCT00256230	I/II	7	Completed
		Glioblastoma	NCT03034135	II	24	Completed

### 4.1 Metal chelating agents that can chelate copper in CRC treatment

Copper chelators are compounds that bind to monovalent or divalent copper and play a crucial role because of the lack of free copper ions in the body and the varying affinity of copper ions for proteins. These chelators modulate the redox activity of copper and have the potential to redistribute copper between proteins.^82^, ^166^ There are many types of copper chelators, including polyaminocarboxylates, acyclic amino, macrocyclic amino, D‐penicillamine, thiomolybdate, hydroxyquinoline chelators, dithiocarbamates, diamines and cuprizones.^166^ Among them, the most studied copper chelators for CRC mainly include TM, D‐penicillamine, trientine, bathocuproinedisulphonic acid, and N, N, N’, N’‐tetrakis‐[2‐pyridylmethyl]‐ethylenediamine (TPEN). Extensive studies have established the crucial role of TM in overcoming chemoresistance in CRC. Specifically, TM augments the cytotoxic effects of chemotherapeutic agents such as oxaliplatin, irinotecan, and 5‐fluorouracil (5‐FU) on CRC cells. Notably, TM enhanced oxaliplatin sensitivity by upregulating CTR1 expression in DLD1 and SW620 cells, which, in turn, increased copper ion entry into the cells, thereby promoting the killing of CRC cells.^167^ With irinotecan and 5‐FU, TM impeded CRC metastasis by reducing angiogenesis in 24 patients with metastatic CRC, primarily by a reducing relevant factors, including VEGF, IL‐8 and IL‐6.^168^ Additionally, TM was effective against BRAF‐mutated CRC cells, inhibiting cell proliferation by targeting the Ras‐RAF‐MEK‐ERK signalling pathway and reversing the chemoresistance of BRAF V600E CRC cells.^169^ Notably, D‐penicillamine and bathocuproinedisulphonic acid have analogous functions to TM.^170^ Furthermore, TPEN can induce HCT116 cell death through redox cycling and ROS generation.^171^ When combined with 5‐FU, TPEN enhanced apoptosis by increasing ROS production and Mcl‐1 ubiquitination in CRC cell lines, thereby augmenting anticancer activity and overcoming chemoresistance.^172^

Other copper chelators such as trientine have also shown promise in CRC treatment. When combined with methotrexate, trientine inhibits CRC development and angiogenesis and is a potential new strategy for CRC treatment.^173^ However, current copper chelators are associated with a series of toxic side effects owing to their chelation of various other metal ions.^174^, ^175^ Shi et al. identified a novel copper chelator, N‐(2‐(pyridin‐3‐yl)thieno[3,2‐c]pyridine‐4‐yl)acetamide (JYFY‐001). This chelator enhanced the efficacy of programmed cell death protein 1 (PD‐1) inhibitors, increased apoptosis in HCT116 and SW620 cells and impaired glucose metabolism in these cells.^176^ In summary, the primary function of copper chelators in CRC is to inhibit cell proliferation and promote cell death. Importantly, these effects are particularly pronounced in drug‐resistant CRC cells, addressing a major challenge in clinical CRC therapy.

### 4.2 Copper ionophores in CRC treatment

Copper ionophores primarily function by dysregulating metal flux and increasing metal import beyond the metal export capacity of cells.^177^ Unlike copper chelators, which mainly inhibit cuproplasia, copper ionophores predominantly induce cuproptosis and play a distinct role in cancer therapy.^178^ Many copper ionophores exist, including elesclomol (ES), DSF, diethyldithiocarbamate, diacetylbis (N4‐methylthiosemicarbazone) and glyoxal‐bis(N4‐methylthiosemicarbazone).^178^ Among these, ES and DSF have been the most extensively studied in CRC. ES is an important copper ionophore in cancer therapy that induces cytotoxicity by transporting Cu^2+^ into cells through the formation of ES‐Cu complexes. Once inside the cell, copper ions can lead to cancer cell apoptosis by inducing oxidative stress,^179^ or target the TCA cycle proteins and FDX1, inducing cell death through cuproptosis. ES alone cannot cause cuproptosis; however, it mainly promotes apoptosis by increasing ROS levels and activating caspase enzymes.^49^, ^180^ However, recent studies have identified several potential targets. In CRC, high ATP7A and ATP7B expression contributes to oxaliplatin resistance.^181^, ^182^, ^183^ ES countered this resistance by degrading ATP7A, causing copper retention and ROS accumulation, which in turn enhanced ferroptosis.^44^ Furthermore, 4‐OI boosts cuproptosis by targeting GAPDH to inhibit aerobic glycolysis in CRC cells. The combination of elesclomol‐Cu and 4‐OI improved antitumour effects in CRC cells.^161^

DSF is another copper ionophore that effectively induces CRC cells death. DSF, a derivative of thiuram, was approved by the United States Food and Drug Administration in 1951 for the treatment of alcohol dependency.^184^ Its therapeutic efficacy is attributed to the irreversible inhibition of aldehyde dehydrogenase (ALDH) activity.^185^ However, emerging evidence has revealed anticancer properties of DSF. DSF and its primary metabolite, diethyldithiocarbamate, induce ROS accumulation and trigger apoptosis.^186^, ^187^, ^188^ Given that ALDH serves as an important biomarker for cancer stem cells and plays a pivotal role in tumour progression, the targeting of ALDH by DSF results in diminished tumourigenic potential.^189^ Furthermore, DSF significantly suppresses the expression of stemness‐related transcription factors, such as SOX2, Nanog and OCT3/4.^190^ In terms of tumour‐related signalling pathways, existing studies have demonstrated that DSF can attenuate the activity of NF‐κB, effectively inhibiting osteoclast differentiation.^191^ Chiba et al. confirmed that DSF exerts its antitumour effects by activating p38, thereby modulating the mitogen‐activated protein kinases MAPK signalling pathway.^192^ As both a proteasome inhibitor and divalent metal ionophore, DSF, through its active sulphhydryl groups, can inhibit the 26S proteasome, thereby impeding the ubiquitin‐proteasome system in a copper ion‐dependent manner.^193^ Upon binding to copper, it induces tumour cell death. This anticancer activity is primarily mediated by the formation of diethyldithiocarbamate‐copper complex (CuET), a compound resulting from the interaction between DSF metabolites in vivo and copper ions.^194^ In CRC, the primary molecular mechanisms include CuET‐mediated reduction of aerobic glycolysis via the miR‐16‐5p and 15b‐5p/ALDH1A3/PKM2 axes.^195^ Disulphiram/copper complex targets ULK1 to induce autophagy,^196^ induces immunogenic death to inhibit proliferation,^197^ and synergistically enhances its cytotoxic effects in the pH‐acidic TME by altering cell metabolism, Akt kinase and NF‐κB activity and increasing ROS production in CRC cells.^198^ During chemotherapy for CRC, DSF/Cu amplifies the efficacy of gemcitabine by inhibiting NF‐κB activity, and similarly enhances the effects of 5‐FU.^199^, ^200^ In other digestive tract tumours, such as hepatocellular carcinoma, DSF/Cu triggers ER stress by altering ER structures and raising Ca^2+^ levels. This effect is coupled with GSH depletion, heightened lipid peroxides, and a compensatory increase in solute carrier family 7 member 11 and activated expression of ATF4, thereby inducing both ferroptosis and cuproptosis.^43^, ^201^ Moreover, the encapsulation of DSF in nanoparticles or liposomes has been reported to augment its therapeutic effects.^202^, ^203^ Currently, studies on copper ionophores in CRC mainly focused on DSF and ES, and other copper ionophores need to be further explored to enhance their therapeutic effect on CRC.

### 4.3 Copper‐based complexes and nanoparticles in CRC treatment

Since cancer and normal cells respond differently to copper, copper complexes have been developed as potential anticancer agents. Copper compounds have attracted interest because of their specific therapeutic properties targeting particular sites.^204^ These copper‐based compounds affect the function of critical cellular organelles, including the mitochondria and endoplasmic reticulum, thereby disrupting their function and ultimately leading to cell death.^205^ In CRC treatment, copper‐based compounds are the most extensively researched form of copper intervention. These predominantly include ligands, liposomes and nanoparticles. The following sections summarise and categorise these developments: copper‐based complexes mainly include copper (I) complexes, copper (II) complexes and ternary copper (III) complexes, and copper (II) is mainly used for these complexes due to its stability. Briefly, these complexes exhibit anticancer properties primarily by inhibiting the proliferation of CRC cells.^206^, ^207^ The key molecular mechanisms involve the inhibition of topoisomerase IIa activity^208^ and interaction with DNA to induce double‐stranded pDNA cleavage.^209^ Additionally, copper‐based complexes also promote cancer cell apoptosis through various mechanisms,^210^, ^211^ including influencing ROS production,^212^, ^213^, ^214^, ^215^ inhibiting the activity of the NF‐κB^215^ and JAK2/STAT5 signalling pathway,^216^ activating the p53 signalling pathway,^217^, ^218^, ^219^ and activating caspase‐related proteins.^220^ Moreover, copper‐based complexes can reverse chemoresistance in CRC treatment, particularly against drugs such as oxaliplatin, cisplatin and doxorubicin. In conjugation with oxaliplatin, copper‐based complexes inhibit the activity of the 26S proteasome, leading to the accumulation of polyubiquitinated proteins, suppression of the ubiquitin‐proteasome pathway, and triggering of endoplasmic reticulum stress.^221^ Notably, cisplatin itself induces apoptosis through mechanisms such as caspase‐3/7 activation, p53 induction and Poly(ADP‐ribose) polymerase cleavage, whereas copper‐based complexes induce apoptosis in CRC cells.^36^ For doxorubicin, these complexes trigger cell death via apoptosis, as evidenced by increased BAX protein expression relative to BCL‐2, depolarisation of the mitochondrial membrane potential, and autophagy.^222^ Therefore, copper‐based complexes show great potential for CRC therapy, primarily by inducing immunogenic cell death.

In addition to copper‐based complexes, copper‐based nanoparticles have shown promising advancements in antitumour applications. In CRC treatment using nanomedicines, delivering lethal doses of copper to cancer cells is crucial for the induction of cell death. The use of inorganic nanoparticles for copper delivery is a viable strategy. Recently, our group developed two advanced CRC treatment strategies specifically for patients with low‐lying rectal cancer following radiotherapy. We designed a copper‐ion‐loaded oxygen generator and sodium alginate hydrogel containing elesclomol‐Cu and galactose. Our study indicated that an increased concentration of copper ions in CRC cells could efficiently trigger cuproptosis. Furthermore, these innovative treatments counteracted the radiotherapy‐induced upregulation of PD‐L1 expression. Consequently, these novel therapeutic strategies exhibit promising potential for treating CRC by promoting cuproptosis and reversing tumour immune escape.^223^, ^224^ Chang et al. highlighted the development of release‐based nanomedicine by designing a core‐shell nanostructure composed of Cu_2_O@CaCO_3_, which was further modified with hyaluronic acid. This design aims to provide synergistic therapies targeting CRC and triggered by the TME, including photothermal, photodynamic, chemodynamic and calcium overload‐mediated approaches.^225^ Through biomimetic modifications, the blood circulation and tumour targeting abilities of these nanomedicines were significantly enhanced. The underlying mechanisms primarily involve the induction of apoptosis and autophagy in CRC cells through the upregulation of Bax, p53 and caspase expression.^226^, ^227^, ^228^, ^229^, ^230^ Zhou et al. revealed that microwave dynamic therapy mediated by copper‐based nanoparticle could improve cancer treatment by inducing ferroptosis in CRC cells.^48^ Furthermore, copper‐based nanomedicines can suppress distant metastasis and recurrence of CRC by inhibiting COX‐2 expression, reprogramming macrophages from M2 phenotype to M1 phenotype, and stimulating an immune response akin to vaccination upon primary tumour elimination.^225^, ^231^ For addressing CRC chemoresistance, copper‐based nanomedicines could be used as drug delivery system for oxaliplatin and 5‐FU‐resistant CRC cells.^232^, ^233^ In mice models, a copper polypyridine complex encapsulated in natural nanocarriers triggered autophagy‐dependent apoptosis, exhibiting potent tumour growth inhibition.^234^ In conclusion, copper‐based compounds are primarily utilised in the treatment of CRC by inducing cell death and exhibit notable efficacy in chemotherapy‐resistant situations. However, these compounds predominantly target divalent copper ions. The stabilisation of monovalent copper ions and the development of compounds that minimise intracellular reduction while augmenting their therapeutic effects are potential research directions.

### 4.4 Radioisotope ^64^Cu in CRC treatment

Copper‐64 (^64^Cu) is a radioisotope with a half‐life of approximately 12.7 h, which can be simultaneously used for both positron emission tomography (PET) imaging and potential therapy due to its specific decay properties (β^+^ and β^−^). Emerging evidence supports the use of ^64^Cu radio‐pharmaceuticals for PET imaging and treatment of hypoxic (low tissue oxygenation) solid tumours. The diagnostic accuracy of CRC has been greatly improved by ^64^Cu, which not only enhances the uptake of radioactive copper in CRC lesions in vivo^235^, ^236^, ^237^ but is also a promising PET tracer for noninvasive imaging of VEGF expression in CRC xenografts.^238^ Additionally, it facilitates the detection of small CRC lesions in the pelvic and abdominal cavities, enabling precise assessment of CRC infiltration in a noninvasive manner.^239^

In addition to its imaging capabilities, ^64^Cu significantly improves cell death and reduces chemo‐resistance in CRC. Studies have shown that ATOX1 and wild‐type p53 promote the entry of ^64^Cu into the nucleus, thereby enhancing its cytotoxic effects on CRC cells.^240^ In addition, copper‐67 (^67^Cu) also plays an important role in advancing CRC radio‐immunotherapy by synergistically enhancing the eradication of CRC cells and metastatic liver foci.^241^, ^242^ When conjugated to platinum, ^64^Cu showed extreme efficacy in destroying CRC cells.^243^, ^244^ Furthermore, when ^64^Cu was conjugated to cetuximab, it killed both CRC cells and wild‐type KRAS CRC cells.^245^, ^246^ Therefore, radioisotope ^64^Cu has shown promising applications in the theranostics of CRC.

## 5 FUTURE PERSPECTIVES AND CONCLUSIONS

Copper, as a trace element, plays a crucial role in copper homeostasis, signalling pathways and cellular metabolism, which in turn influence cell proliferation, angiogenesis and distant metastasis in numerous cancers. Disruption of copper homeostasis can lead to excess copper accumulation, resulting in elevated ROS levels and promotion of proliferation, a process known as cuproplasia. Conversely, an excessive copper levels in the body can trigger cuproptosis. Therefore, it is vital to maintain a balanced fluctuation of copper ions in the body.

This review investigated the role of copper ions in CRC by focusing on two primary aspects. First, excess copper ions promote CRC proliferation, metastasis, angiogenesis and antitumour immune response, primarily through mechanisms such as the activation of the NF‐κB signalling pathway, CyclinD1 transcriptional activation, angiogenesis promotion, and EMT. Second, elevated copper ion concentrations can trigger cuproptosis, which is usually higher in CRC tissues than in neighbouring tissues. Therefore, this distinction is crucial for targeted copper ion therapy for CRC, which currently involves copper ion chelates, ionophores and compounds. These therapies target copper ions to inhibit cancer cell proliferation or induce cell death. Additionally, radioisotope ^64^Cu is significant for the theranostics of CRC. This review not only highlights the functional roles and mechanisms of copper ions in physiological states, providing valuable insights for future research, but also underscores the role of the intestinal tract in the absorption and utilisation of copper ions, offering theoretical support for future CRC treatments. Current CRC treatments targeting copper ions are notable, particularly considering the elevated serum copper levels (mainly detected by plasma CP levels) in patients with CRC, which could facilitate noninvasive, real‐time monitoring of CRC progression and recurrence. However, the molecular mechanisms underlying the effects of copper on CRC remain unclear. A key challenge is effectively differentiating between cuproplasia and cuproptosis in the human body. The development of reliable methods to quantify total copper levels in plasma and cells, including their labile forms and bioavailable, is critical for the creation of companion diagnostics. These diagnoses help identify patient populations suitable for copper‐targeted therapy. Additionally, evaluating patients’ functional ‘copper status’ is imperative to reduce adverse effects and assess treatment effectiveness. Furthermore, it remains critical to determine whether there are differences in the range of copper concentrations for cuproplasia and cuproptosis mutations in different cancer types.

In the future, homeostasis research will be poised for significant breakthroughs, especially in converting insights from copper homeostasis mechanisms into effective CRC treatments. From a molecular perspective, the investigation of copper‐dependent signalling pathways, along with the control of cuproplasia and cuproptosis via copper‐centric molecular mechanisms, is the most promising area of study. Given the significant impact of immunotherapy on CRC treatment in recent years, combined with extensive bioinformatics research on the role of copper ions in CRC, future studies should focus on exploring the interactions between copper ions and the TME and their effects on immune responses. This includes investigating the multiomics characteristics of CRC under specific conditions such as different microsatellite stability statuses, resistance to drug treatments, patients with KRAS gene mutations, and variations in copper ion levels in tissues. By analysing of a large number of clinical patient samples, this research will provide a deeper theoretical and practical foundation for future immunotherapy strategies targeting copper ions.

In clinical trials, strategies for treating tumours by targeting copper ions have primarily focused on the use of approved copper chelators (e.g. TM) and copper ionophores (e.g. DSF). Although these agents have demonstrated the potential to inhibit tumour growth in existing clinical models, there is a need to more precise identification of patient populations that would benefit from them, particularly in terms of specifying the types of tumours and understanding the genetic background of the patients. From a clinical perspective, copper is an important trace element involved in numerous biological processes, and comprehensive inhibition mediated by targeting copper can negatively affect normal cellular functions, especially during long‐term treatment. Additionally, determining the correct dosage and the bioavailability of drug is challenging and requires a balance between effective antitumour activity and reduced toxicity to normal tissues. Finally, clinical experiments targeting copper ions will also require the development of biomarkers to predict and monitor copper levels and their bioactivity, as well as the development of combination therapies with other therapeutic modalities to improve efficacy and reduce adverse effects on normal tissues.

Notably, copper nanoparticles represent a potential anticancer material with diverse applications. As a drug carrier, they can enhance the efficiency of drug delivery into tumour cells, increase therapeutic specificity and reduce damage to healthy cells. Utilising their optical properties, copper nanoparticles can kill cancer cells by generating local high temperatures or ROS under laser irradiation. Moreover, these treatments can enhance the efficacy of chemotherapy and radiotherapy. Despite these advantages, the application of copper nanoparticles faces several challenges, including ensuring biocompatibility, controlling toxicity, improving tumour targeting, and studying their metabolism and clearance mechanisms to prevent potential side effects. Future research should focus on improving the design of copper nanoparticles by adjusting their size, shape and surface modifications to enhance therapeutic outcomes; developing multifunctional copper nanoparticles that integrate drug delivery, photothermal, and photodynamic therapy functions for a unified treatment approach; and designing personalised treatment systems for specific tumour types or patients by integrating genomic and proteomic data.

**FIGURE 5:**
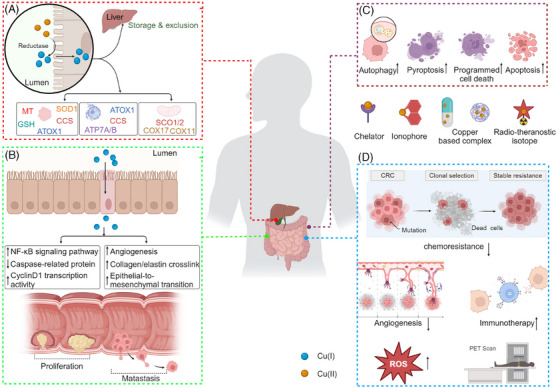
**The schematic illustration delineates the multifaceted role of copper ions in CRC malignancy, alongside their therapeutic and diagnostic applications based on copper**. (A) The normal transport mechanisms of copper ions within the body and their utilisation at the intracellular level. (B) The detailed molecular mechanisms through which copper ions contribute to CRC proliferation and metastasis. (C) The mode of cell death induced by copper ion toxicity. (D) The mechanisms by which copper ions are involved in the treatment and diagnosis of CRC.

Therefore, in this review, acknowledging the intestine as the primary site for copper ion absorption and CRC as the most common type of cancer within the gastrointestinal tract, we systematically explored the physiological roles and utilisation processes of copper ions in the body. Considering the narrow physiological range of copper ion concentrations, this review comprehensively assessed the role of copper ions in mediating disease under pathological conditions, and clarified how abnormal copper ion concentrations drive CRC proliferation and metastasis. Furthermore, this review outlined the role of targeted copper ion strategies in CRC diagnosis and treatment, emphasising the cytotoxic effects of copper ions inducing multiple forms of cell death as well as mechanisms for reversing chemoresistance, reducing angiogenesis, enhancing immunotherapy and increasing ROS levels (Figure 5). Through this comprehensive summary, we aim to deepen our understanding of the physiological roles of copper ions and their mechanisms of action in CRC, providing a theoretical foundation for the future diagnosis and treatment of CRC and other malignancies.

## AUTHOR CONTRIBUTIONS

YL, LCG and KY designed and conducted this review. KY critically revised the final version of the manuscript. YHW conceived and drafted this manuscript. YHW and PP drew the figures. YHW drew the tables. All the authors approved the final version of the manuscript.

## CONFLICT OF INTEREST STATEMENT

The authors declare that they have no known competing financial interests or personal relationships that could have appeared to influence the work reported in this paper.

## ETHICS STATEMENT

Not applicable.
